# Comparison of Postoperative Unfavorable Events in Patients with Low-Risk Papillary Thyroid Carcinoma: Immediate Surgery Versus Conversion Surgery Following Active Surveillance

**DOI:** 10.1089/thy.2022.0444

**Published:** 2023-02-14

**Authors:** Takahiro Sasaki, Akira Miyauchi, Makoto Fujishima, Yasuhiro Ito, Takumi Kudo, Takuya Noda, Tsutomu Sano, Taketoshi Kishi, Tomohiko Nakamura

**Affiliations:** ^1^ Department of Head and Neck Surgery, Kuma Hospital, Kobe, Japan.; ^2^ Department of Surgery, Kuma Hospital, Kobe, Japan.; ^3^ Department of Internal Medicine, Kuma Hospital, Kobe, Japan.

**Keywords:** active surveillance, conversion surgery, papillary microcarcinoma, prognosis, thyroid, unfavorable events

## Abstract

**Background:**

Active surveillance (AS) for low-risk papillary thyroid microcarcinoma (PTMC) was initiated at Kuma Hospital in 1993 and has gradually spread worldwide. We previously demonstrated that AS is associated with a much lower incidence of unfavorable events than immediate surgery (IS). However, conversion surgery (CS) raises concerns about increased surgical complications due to advanced disease. In this study, we conducted a comparative analysis of unfavorable events after IS and CS.

**Methods:**

Between 2005 and 2019, 4635 patients clinically diagnosed with low-risk PTMC at Kuma Hospital were enrolled. Of these, 2896 underwent AS (AS group), and the remaining 1739 underwent IS (IS group). To date, 242 patients (8.4%) in the AS group have undergone CS for various reasons (CS group).

**Results:**

The incidence of unfavorable events, such as levothyroxine administration after surgery, postoperative hematoma, transient/persistent hypoparathyroidism, and transient/persistent vocal cord paralysis, did not differ between the CS and IS groups. None of the patients in the CS group had permanent vocal cord paralysis; however, this occurred in 15 patients (0.9%) in the IS group and was caused by accidental injury in 4 patients and carcinoma invasion in 11 patients. The incidence of surgery, levothyroxine administration, postoperative hematoma, transient/permanent hypoparathyroidism, and vocal cord paralysis was significantly higher (*p* < 0.001) in the IS group than in the AS group. There were no differences in the incidence of lymph node recurrence and overall mortality between the AS and IS groups. None of the patients in the AS and IS groups showed distant metastasis or died from thyroid carcinoma.

**Conclusions:**

There were no differences in the incidence of unfavorable events between the CS group and the IS group. Although none of the CS and AS groups had permanent vocal cord paralysis, accidental injury of the recurrent laryngeal nerve occurred in four patients (0.2%) in the IS group. The IS group had a significantly higher incidence of unfavorable events than the AS group. The prognoses of patients in both the AS and IS groups were excellent. Therefore, we recommend AS as the first-line management for low-risk PTMC.

**Correction added** on February 20, 2023 after first online publication of November 9, 2022: In the Methods section of the abstract, *242 patients (0.8%)* has been corrected to *242 patients (8.4%)*.

## Introduction

Recently, there has been growing scientific interest in the management of low-risk papillary thyroid microcarcinoma (cT1aN0M0, PTMC).^1^ It is well known that PTMC shows favorable outcomes after surgery. In our series, the 5- and 10-year lymph node recurrence-free survival rates, distant recurrence-free survival rates, and cause-specific survival rates were 99% and 99%, 100% and 100%, and 100% and 100%, respectively.^2^ However, surgery may induce significant unfavorable events, such as vocal cord paralysis, hypothyroidism, hypoparathyroidism, and neck discomfort.

Although surgery for PTMC is not technically difficult, Oda et al reported that postoperative hematoma, permanent vocal cord paralysis, and permanent hypoparathyroidism occurred in 0.5%, 0.2%, and 1.6% of patients, respectively, who underwent surgery by well-experienced surgeons at Kuma Hospital.^3^

Active surveillance (AS) for PTMC, initiated in Kuma Hospital in 1993 and Cancer Institute Hospital in 1995, has become accepted worldwide as a management strategy and was endorsed by the guidelines of the Japan Association of Endocrine Surgery and American Thyroid Association.^4–6^ Patients who undergo AS may avoid various surgical complications, and oncological outcomes are excellent.^7–15^ However, some patients show disease progression, such as tumor enlargement and the appearance of novel node metastasis requiring conversion surgery (CS). There are concerns that CS may be associated with higher surgical complications than immediate surgery (IS) because of advanced disease. Therefore, we conducted a comparative study of unfavorable events among patients who underwent IS and CS.

## Materials and Methods

### Patients

Between 2005 and 2019, 4635 patients were clinically diagnosed with low-risk PTMC. All patients had a cytological diagnosis of Bethesda V or VI and were aged ≥20 years. None of the patients showed highly aggressive cytology, such as a tall cell variant. Tumor status was evaluated using ultrasound examination, and other imaging studies were performed if indicated. None of the patients had high-risk features, such as lymph node metastasis, distant metastasis, significant extrathyroidal invasion, or vocal cord paralysis; therefore, they were classified as cT1aN0M0 under the TNM staging system.^16^

Of the 4635 patients, 2896 (62.5%) chose AS (AS group), and the remaining 1739 (37.5%) underwent IS within one year after the diagnosis (IS group). Total thyroidectomy was performed in 768 patients (44.2%) in the IS group and 118 patients (48.8%) in the CS group. There was no significant difference (*p* = 0.18) in the extent of thyroidectomy between the two groups. The reasons for IS included close location of the tumor to the recurrent laryngeal nerve or trachea, although classified as cT1a,^17,18^ as well as patient preferences. Although these patients had cT1aN0M0 tumor, in 114 patients (6.6%) invasion of the surrounding tissues, including the recurrent laryngeal nerve, or metastasis to lateral lymph nodes was observed during surgery.

**FIG. 1.**
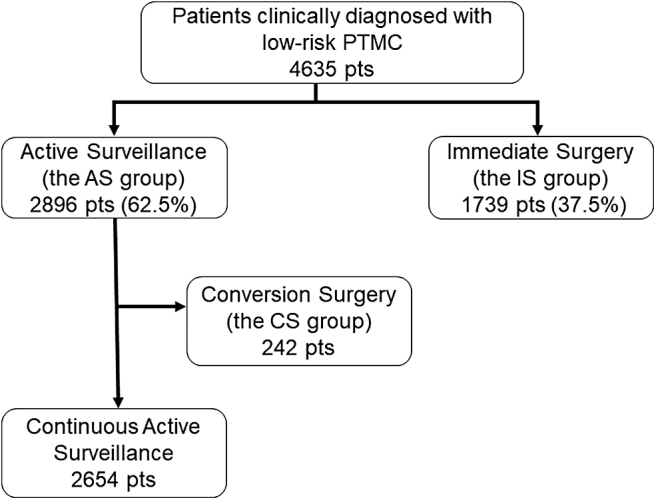
A flowchart of patients clinically diagnosed with low-risk PTMC. PTMC, papillary thyroid microcarcinoma.

These features were not present in the remaining 1625 (93.4%) patients. In the AS group, 242 patients (8.4%) underwent CS 1 year or longer after AS because of disease progression (tumor enlargement by at least 3 mm in 52 patients, appearance of lymph node metastasis in 17 patients, and both in 3 patients), associated thyroid or parathyroid disease (55 patients and 6 patients, respectively), or a change in patient or physician preference (47 patients and 62 patients, respectively) (CS group). The median period from the initiation of AS to CS was 2.8 years (range 1.0–11.3 years). Figure 1 shows the flowchart of patients clinically diagnosed with low-risk PTMC.

Table 1 gives the background and clinical characteristics of the patients in the CS and IS groups. The age at diagnosis was lower in the CS group (*p* < 0.001) than that in the IS group; however, patient age at surgery did not differ between the two groups. The incidence of coexisting Graves' disease was higher (*p* = 0.011), and multiple tumors were more frequent (*p* = 0.002) in the CS group than in the IS group. In this study, the initial tumor size at diagnosis was set as the average tumor size at the first and second ultrasound examinations to minimize observer variation.

**Table 1.** Comparison of Background and Clinical Characteristics Between the Patients in the Conversion Surgery Group and the Immediate Surgery Group

Variables	CS group (*N* = 242)	IS group (*N* = 1739)	*p*
Male/female sex	29/213 (88.0)	193/1546 (88.9)	0.683
Age at diagnosis, years	53 (21–80)	55 (20–92)	<0.001
Age at surgery, years	55 (22–84)	55 (20–92)	0.660
Hashimoto disease^a^	90 (37.2)	553 (31.8)	0.093
Graves' disease	30 (12.4)	132 (7.6)	0.011
Family history^b^	6 (2.5)	61 (3.5)	0.407
Multiplicity^c^	44 (18.2)	198 (11.4)	0.002
Initial tumor size at diagnosis, mm^d^	7.5 (3.0–10.0)	8.0 (2.5–11.0)	0.006
Tumor size at surgery, mm	9.0 (2.0–19.0)	8.0 (2.5–18.0)	<0.001
Extent of thyroidectomy			
Total/hemithyroidectomy	118/124 (48.8)	768/971 (44.2)	0.178
Central compartment dissection	228 (94.2)	1731 (99.5)	<0.001
Lateral compartment dissection	14 (5.8)	8 (0.5)	<0.001

Values are median (ranges), or number (proportion %).

The tumor in the patient in the IS group measured 10 and 12 mm at the first and second examinations, respectively.

^a^ Patients who were positive for antithyroglobulin antibody and/or TPO antibody and not diagnosed with Graves' disease.

^b^ One or more first-degree relatives had papillary thyroid carcinoma.

^c^ Evaluated by imaging studies.

^d^ The average of the tumor sizes at the first and the second examinations was considered the baseline size to minimize observer variations.

CS, conversion surgery; IS, immediate surgery; TPO, thyroid peroxidase antibody.

The initial tumor size at diagnosis was significantly smaller (*p* = 0.006) in the CS group than that in the IS group, although tumor size at surgery was larger (*p* < 0.001). The extent of thyroidectomy did not differ between the two groups. However, lateral compartment dissection was more frequent in the CS group (*p* < 0.001) than that in the IS group.

This study was approved by the ethics committee of Kuma Hospital (No. 20200709-1).

### AS for PTMC

AS was performed in patients who were diagnosed with low-risk PTMC and chose this management, as described previously.^7,19,20^ Our AS of clinically low-risk PTMC was a management plan for performing CS at an appropriate time in cases of disease progression. In brief, we asked patients to visit our clinic periodically to evaluate tumor status and nodal status on ultrasonography. We regarded tumors as enlarged when the maximal diameter increased by ≥3 mm compared with the initial size. We discussed CS with the patients, and if the patients preferred to pursue AS, we continued AS until the tumor size reached 13 mm. If lymph node metastasis was suspected, we performed cytological examination of the node with thyroglobulin measurement of the needle washout. We recommended CS if a nodal metastasis was diagnosed.

### Definition of vocal cord paralysis and hypoparathyroidism after surgery

Laryngoscopy was routinely performed before and two or three days after surgery. None of the patients in our series had preoperative vocal cord paralysis due to carcinoma invasion of the recurrent laryngeal nerve. For patients with postoperative vocal cord paralysis, regular monitoring by laryngoscopy was performed. If paralysis continued 1 year after surgery, we regarded the patients as having permanent vocal cord paralysis, and if this recovered within 1 year, patients were classified as having had temporary vocal cord paralysis.

Patients requiring vitamin D (calcitriol, alfacalcidol, or eldecalcitol) and/or calcium preparations after surgery were considered to have hypoparathyroidism. We interviewed all patients to determine whether they were taking low-dose calcium supplements or whether they had prescriptions for vitamin D preparations from other hospitals, taking care not to duplicate prescriptions. Patients who were already taking vitamin D/calcium before surgery for the treatment of other diseases were considered to have postoperative hypoparathyroidism only if an increase in dosage was required. If either or both of these medications were continuously administered one year after surgery, patients were considered to have permanent hypoparathyroidism. If the drug could be discontinued within one year, temporary hypoparathyroidism was diagnosed.

### Postoperative follow-up

After surgery, patients were asked to attend our hospital for blood tests and ultrasound examinations at least once per year. For patients referred to other hospitals, questionnaires were sent yearly to evaluate their condition. When suspicious lymph nodes were detected by ultrasonography, we performed fine-needle aspiration for cytology and thyroglobulin measurement in the needle washout. If either of these were positive, nodal recurrence was diagnosed.

### Statistical analysis

StatFlex software (Artec, Osaka, Japan) was used to perform the univariate and multivariate analyses. The chi-square test was used to compare the variables. The Kaplan–Meier method and log-rank test were used to calculate overall mortality. Statistical significance was set at *p* < 0.05.

## Results

First, we compared unfavorable events that occurred in 242 patients in the CS group and 1739 patients in the IS group (Table 2). There were no differences in the incidence of unfavorable events, such as levothyroxine administration after surgery, postoperative hematoma, transient/persistent hypoparathyroidism, and transient/persistent vocal cord paralysis, between the two groups. Although none of the patients in the CS group had permanent vocal cord paralysis, 15 patients (0.9%) in the IS group acquired vocal cord paralysis. Of these cases, 4 (0.2%) were caused by accidental injury (2 accidental ligations, 1 accidental transection, and 1 accidental heat injury).

**Table 2.** Incidences of Unfavorable Events After Conversion Surgery and Immediate Surgery

Variables	CS group (*N* = 242)		IS group (*N* = 1739)		*p*
	No. of events	% [CI]	No. of events	% [CI]	
Levothyroxine administration after surgery^a^	154	63.6 [57.9–70.0]	1134	65.2 [63.0–67.5]	0.631
Postoperative hematoma requiring reoperation	1	0.4 [0.1–2.9]	13	0.7 [0.4–1.3]	0.561
Transient vocal cord paralysis	26	10.7 [7.5–15.4]	151	8.7 [7.5–10.1]	0.292
Permanent vocal cord paralysis due to^b^	0	0.0 [0–NaN]	15	0.9 [0.5–1.4]	0.147
Accidental transection or injury			4	0.2 [0.1–0.6]	
Surgery for tumor invasion			11	0.6 [0.4–1.1]	
Transient hypoparathyroidism	60	24.8 [19.9–30.9]	362	20.8 [19.0–22.8]	0.157
Permanent hypoparathyroidism^c^	5	2.1 [0.9–4.9]	24	1.4 [0.9–2.1]	0.405
Recurrence in the neck					
Contralateral lobe	0	0.0 [0–NaN]	6	0.3 [0.2–0.8]	0.360
Lymph node	1	0.4 [0.1–2.9]	3	0.2 [0.1–0.5]	0.434

^a^ Patients who were administered levothyroxine before surgery were not included.

^b^ Persistent vocal code paralysis on laryngoscopy one year after surgery or longer.

^c^ Administration of vitamin D and/or calcium preparation one year after surgery or longer.

[CI], confidence interval; NaN, not a number.

In the remaining 11 patients, tumor invasion of the recurrent laryngeal nerves was found during surgery, requiring shaving or resection of the nerves, although they were classified as cT1a preoperatively. Reconstruction of the recurrent laryngeal nerve was performed in all 13 patients who underwent nerve resection or transection. The patients achieved recovery in phonation; however, the vocal cords on the side remained immobile. The recurrence of carcinoma after surgery was also an unfavorable event. The recurrence rate in the contralateral lobe and neck lymph nodes did not differ significantly between the groups.

Next, we compared unfavorable events according to the management of intention to treat at the initial diagnosis. Table 3 gives the overall incidence of unfavorable events in patients who chose AS and in those who underwent IS. The incidence of surgery, levothyroxine administration, postoperative hematoma, transient and permanent vocal cord paralysis, transient and permanent hypoparathyroidism, and recurrence in the contralateral thyroid lobe were significantly higher in the IS group than in the AS group. Of the 2896 patients who chose AS, only 242 (8.4%) underwent CS for the following reasons: disease progression (72 patients), physician preference (62 patients), patient preference (47 patients), associated thyroid disease (55 patients), and associated parathyroid disease (6 patients).

**Table 3.** Overall Incidence of Unfavorable Events in Patients Who Chose Active Surveillance and in Those Who Underwent Immediate Surgery

Variables	AS group (*N* = 2896)		IS group (*N* = 1739)		*p*
	No. of events	% [CI]	No. of events	% [CI]	
Surgery	242	8.4 [7.4–9.4]	1739	100. [100–100]	<0.001
Levothyroxine administration after diagnosis^a^	729	25.2 [23.6–26.8]	1134	65.2 [63.0–67.5]	<0.001
Postoperative hematoma requiring reoperation	1	0.0 [0.0–0.2]	13	0.7 [0.4–1.3]	<0.001
Transient vocal cord paralysis	26	0.9 [0.6–1.3]	151	8.7 [7.5–10.1]	<0.001
Permanent vocal cord paralysis caused by^b^	0	0.0 [0–NaN]	15	0.9 [0.5–1.4]	<0.001
Accidental transection or injury			4	0.2 [0.1–0.6]	
Surgery for tumor invasion			11	0.6 [0.4–1.1]	
Transient hypoparathyroidism	60	2.1 [1.6–2.7]	362	20.8 [19.0–22.8]	<0.001
Permanent hypoparathyroidism^c^	5	0.2 [0.1–0.4]	24	1.4 [0.9–2.1]	<0.001
Recurrence in the neck	1	0.0 [0.0–0.2]	9	0.5 [0.3–1.0]	<0.001
Contralateral lobe	0	0.0 [0–NaN]	6	0.3 [0.2–0.8]	0.002
Lymph node	1	0.0 [0.0–0.2]	3	0.2 [0.1–0.5]	0.121
Distant metastasis	0	0.0 [0–NaN]	0	0.0 [0–NaN]	N/A
Death from thyroid cancer	0	0.0 [0–NaN]	0	0.0 [0–NaN]	N/A

^a^ Patients who were administered levothyroxine before diagnosis were not included.

^b^ Persistent vocal code paralysis on laryngoscopy one year after surgery or longer.

^c^ Administration of vitamin D and/or calcium preparation one year after surgery or longer.

AS, active surveillance; N/A, not available.

The disease progression group included 52 patients with increase in tumor size, 17 patients with appearance of node metastasis, and 3 patients with both features. In the IS group, levothyroxine administration was initiated for postoperative hypothyroidism in 1134 patients (65.2%) and in 729 patients (25.2%) in the AS group (*p* < 0.001) to suppress thyrotropin or treat hypothyroidism. None of the patients in the AS group, including those who underwent CS, had permanent vocal cord paralysis, while in four patients (0.2%) in the IS group, accidental injury of the recurrent laryngeal nerve occurred, although all of the procedures were performed by experienced surgeons at Kuma Hospital.

**FIG. 2.**
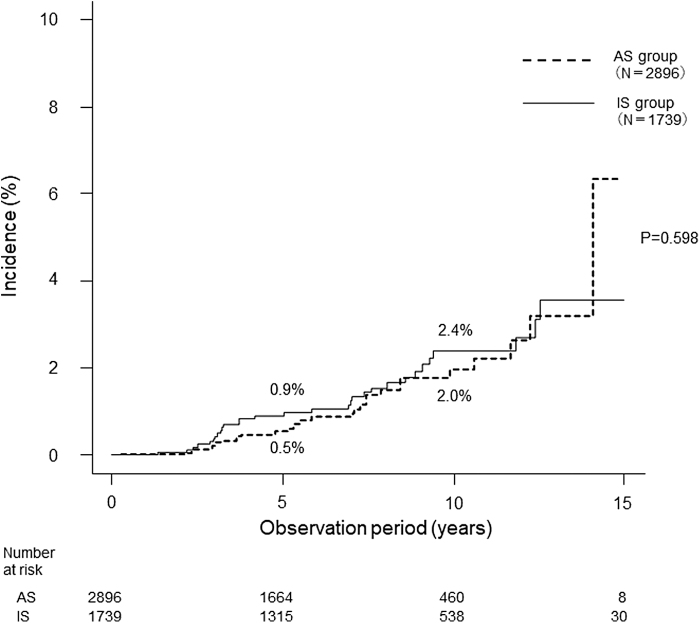
Overall mortality rates of patients in the AS group and the IS group. AS, active surveillance; IS, immediate surgery.

Regarding recurrence in the neck, only one patient (0.03%) in the AS group showed lateral node recurrence after CS due to tumor enlargement. In the IS group, recurrence occurred in the neck in 9 patients (0.5%) (*p* < 0.001), in the remnant thyroid in 6 patients (0.3%), and in the lateral lymph nodes in 3 patients (0.2%). All of the mentioned 10 patients underwent additional surgery. None of the patients developed distant metastases or died from thyroid cancer. The overall mortality rates at 10 years were 2.0% and 2.4% in the AS and IS groups, respectively (not significant) (Fig. 2).

## Discussion

First, we compared the backgrounds, clinical features, and unfavorable events between the IS and CS groups. Compared with the IS group, a larger proportion of patients in the CS group had multiple PTMCs. Patients with multiple PTMCs should undergo total thyroidectomy if operated, indicating that levothyroxine administration becomes mandatory after surgery, and the risk of vocal cord paralysis and hypoparathyroidism significantly increases. Multifocal PTMC is known to be associated with a higher incidence of lymph node metastasis than unifocal PTMC,^21^ although it is not necessarily associated with a poorer prognosis than unifocal PTMC.^7^

Therefore, attending physicians may recommend AS for patients with multifocal PTMC. In the present cohort, 408 patients in the AS group (14.1%) and 198 in the IS group (11.4%) had multifocal PTMC (*p* = 0.008). Although the incidence of coexisting Graves' disease did not differ between the AS and IS groups (191 vs. 132, *p* = 0.198), the CS group included a higher incidence of patients with Graves' disease than the IS group, possibly because patients who had difficulty controlling Graves' disease with antithyroid drugs during AS underwent CS.

In our series, the incidence of unfavorable events, such as levothyroxine administration, postoperative hematoma, transient/permanent vocal cord paralysis, and transient/permanent hypoparathyroidism, in the CS group did not significantly differ from that in the IS group. These findings indicate that delayed surgery did not increase unfavorable surgical events compared with IS.

AS of clinically low-risk PTMC pioneered at our institution is a management plan for performing CS at an appropriate time in cases of disease progression. Therefore, we investigated the difference in the incidence of unfavorable events after AS and IS for patients with PTMC according to the intention to treat. The incidence of unfavorable events, such as levothyroxine administration, postoperative hematoma, transient/permanent vocal cord paralysis, and hypoparathyroidism, was significantly lower in the AS group than that in the IS group, which is consistent with our previous study.^3^ Our hospital specializes in thyroid disease, and all surgeons are experts in thyroid surgery.

Despite this, 4 patients (0.2%) suffered from permanent vocal cord paralysis due to accidental ligation, transection, or thermal injury by electrocautery of the recurrent laryngeal nerve, and 24 (1.4%) patients had permanent hypoparathyroidism in the IS group. If patients underwent surgery performed by nonexperts, the incidence of these events would likely be much higher. This is important when discussing an appropriate management strategy for PTMC. The frequency of unfavorable events did not change between the IS and CS groups, and when comparing the AS and IS groups, it is clear that the IS group had a higher frequency of unfavorable events. Therefore, at Kuma Hospital, we recommend AS as the first-line management for patients with low-risk PTMC.

In the IS group, 11 patients (0.6%) had permanent vocal cord paralysis. We postulate that this was because of the difference in selection criteria between the IS and AS groups. We recommended IS for patients with tumors located on the course of the recurrent laryngeal nerve, although vocal cord paralysis was not detected by fiberoptic laryngoscopy.^17^ In contrast, patients with tumors with no possibility of recurrent laryngeal nerve invasion were selected as candidates for AS; therefore, none of the patients in the CS group experienced vocal cord paralysis caused by tumor invasion. This may indicate that physician selection of patients for IS was appropriate and that physicians made the decision to perform CS at the appropriate time.

In summary, the incidence of unfavorable events in the IS group did not differ from that in the CS group, although it was significantly higher than that in the AS group. Neck recurrence was significantly lower in the AS group than that in the IS group. In addition, the overall mortality rate was similar between the AS and IS groups, suggesting that AS is a preferable first-line management for PTMC.
